# The Durable Chitosan Functionalization of Cellulosic Fabrics

**DOI:** 10.3390/polym15183829

**Published:** 2023-09-20

**Authors:** Sandra Flinčec Grgac, Tea-Dora Biruš, Anita Tarbuk, Tihana Dekanić, Ana Palčić

**Affiliations:** Department of Textile Chemistry and Ecology, University of Zagreb Faculty of Textile Technology, Prilaz baruna Filipovića 28a, HR-10000 Zagreb, Croatia; t.d.birus@gmail.com (T.-D.B.); tihana.dekanic@ttf.unizg.hr (T.D.); ana.palcic@ttf.unizg.hr (A.P.)

**Keywords:** cotton fabric, cotton/polyester blend, chitosan, maleic acid (MA), 1,2,3,4-butanetetracarboxylic acid (BTCA), FTIR-ATR, mechanical properties, whiteness, antimicrobial activity, durability

## Abstract

In this work, the durability of chitosan functionalization of cellulosic textile substrates, cotton and cotton/polyester blended fabrics, was studied. Chitosan is a naturally occurring biopolymer that can be produced inexpensively. It should be dissolved in an acidic solution to activate its antimicrobial and other properties, i.e., good biocompatibility, bioabsorbability, wound healing, hemostatic, anti-infective, antibacterial, non-toxic, and adsorptive properties. The application of chitosan to textile products has been researched to achieve antimicrobial properties, but the durability, after several maintenance cycles, has not. Chitosan functionalization was carried out using maleic acid (MA) and 1,2,3,4-butanetetracarboxylic acid (BTCA) as crosslinking and chitosan-activating agents and sodium hypophosphite monohydrate as a catalyst. To determine durability, the fabrics were subjected to 10 maintenance cycles according to ISO 6330:2012 using Reference detergent 3 and drying according to Procedure F. The properties were monitored after the 3rd and 10th cycles. The crosslinking ability of chitosan with cellulosic fabrics was monitored by Fourier infrared spectrometry using the ATR technique (FTIR-ATR). Changes in mechanical properties, whiteness and yellowing, and antimicrobial properties were determined using standard methods. Compared to maleic acid, BTCA proved to be a better crosslinking agent for chitosan.

## 1. Introduction

Chitosan is one of the most important derivatives of the natural biopolymer chitin found in shells of crustaceans, exoskeletons of insects, cell walls of fungi, and plankton. Its biocompatibility, bioabsorbability, antibacterial, antimicrobial, anticancer, antidiabetic, hemostatic, anti-infective, wound dressing, drug delivery, non-toxic, and adsorptive properties have led to its application in medicine and, more recently, for medical devices. It can also be used to produce hydrogels, foams, scaffolds, etc., with other polymers [1,2,3,4,5,6,7,8,9,10,11,12,13,14,15,16,17,18,19,20,21]. Therefore, research on the use of chitosan in textiles and biomaterials has increased significantly in the last decade [4,5,6,7,8,9,10,11,12,13,14,15,16,17,18,19,20,21,22,23,24,25,26,27,28,29,30,31].

Chitosan is a partially N-deacetylated derivative of chitin, a linear polysaccharide consisting of randomly distributed β-(1→4)-linked d-glucosamine and N-acetyl-d-glucosamine units [2]. Its chemistry is complex, and its properties, i.e., solubility, charge density, and reactivity, depend on several factors, mainly the degree of deacetylation, molecular weight, and pH of the solution. The degree of deacetylation (DDA) refers to the percentage of N-acetyl-d-glucosamine units converted to d-glucosamine units; the higher the DDA, the better the solubility. The higher the molecular weight of chitosan, the higher the viscosity and the lower the solubility. The pH of the solution can affect the charge density of chitosan, with lower pH values leading to higher charge densities due to increased protonation of the amino groups [2,6,7,8,9,10]. However, the pH required for protonation can cause mechanical damage to cellulosic fibers [22].

To be applied to textiles, it must be dissolved in an aqueous acid solution [12,13,14,15,16,17,18,19,20,21,25,26,27,28,29,30,31]. Due to the protonation of the free amino groups, the molecule becomes completely soluble below pH 5. Therefore, acetic acid [5,8,14,27,28], citric acid (CA) [18,19,20,21,29,30], formic acid [8], hydrochloric acid (HCl) [9,15,26], lactic acid [8], 1,2,3,4-butanetetracarboxylic acid (BTCA) [13,16,17,18,30], and, more recently, 2,3-dihydroxy butandionic acid [25] and maleic acid (MA) [31] can be used for its dissolution and activation. When quaternized under acidic conditions, the positive terminal (or free) amino groups of chitosan readily interact with negatively charged bacterial membranes to exert antibacterial activity. When chitosan is applied to cellulose in a quaternized state, it exerts an antibacterial effect that is further enhanced when applied by various techniques such as conjugation, encapsulation, and complexation [32].

As mentioned above, protonation of the chitosan amino groups results in a positively charged polymer that is easier to apply to cellulosic textiles. For the durability of this functionalization, it is necessary to use crosslinking agents that can affect the activity of chitosan. The auxiliaries in textile finishing such as dimethylol dihydroxyethylene urea (DMDHEU) [14], low formaldehyde resins [9], glutardialdehyde (GA) [15], 1,2,3,4-butanetetracarboxylic acid (BTCA) [13,16,18,30], and citric acid (CA) [18,19,20,21,29,30] are the most commonly used crosslinking agents for chitosan. Since glutardialdehyde [27] and formaldehyde [29] are considered toxic and raise health concerns, research is underway for non-toxic crosslinkers, i.e., sodium salts of the tripolyphosphate. In some cases, chitosan hydrochloride is used instead of sodium hypophosphite (SHP) as a catalyst for the finishing agents (BTCA or low-formaldehyde derivatives) [13].

**Figure 1:**
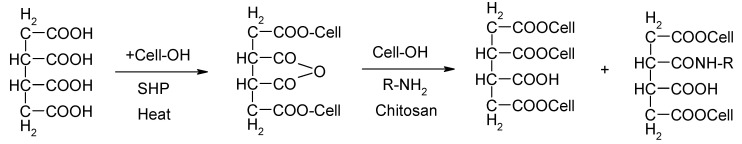
The crosslinking mechanism of BTCA with cellulose in the presence of chitosan.

BTCA has been found to be the most effective crosslinking agent for cotton without significantly affecting the appearance of the textile [16,17,18,19,30,33]. The crosslinking of cellulose with BTCA occurs in several steps (Figure 1). The first step is the formation of a cyclic anhydride that covalently bonds with the hydroxyl groups of cellulose. In this way, a second anhydride is formed from the remaining two carboxyl groups, which in turn reacts with the hydroxyl groups. The reaction ends with the formation of an ester bond between the cellulose and the crosslinking agent [13,17].

**Figure 2:**
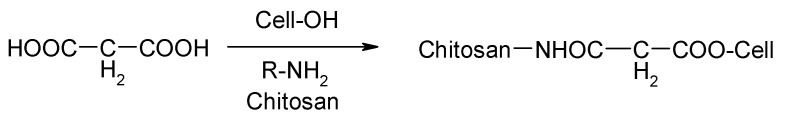
The crosslinking mechanism of MA with cellulose in the presence of chitosan.

Maleic acid (MA) is chemically similar to BTCA and has a carboxylic acid group attached to the adjacent -CH_2_ group in the main molecular chain. Maleic acid is an unsaturated -α,-β-dicarboxylic acid that can only form an ester bond and cannot form secondary cyclic anhydrides with cotton cellulose. The crosslinking mechanism of MA with cellulose in the presence of chitosan is shown in Figure 2. MA is very difficult to homopolymerize by free radicals due to the strong attractive forces between the electrons and steric hindrance by the two carboxyl groups [14,31,34,35,36,37].

For better performance in dyeing and printing polyester fabrics [9,16,38,39,40], some modified chitosans have been developed, e.g., N-[(2-hydroxy-3-trimethyl-ammonium)propyl]chitosan chloride (HTACC) [12,39], or plasma/corona pretreatment has been performed [16,40].

The use of chitosan and/or crosslinking with cellulosic material has been studied, but not durability. There are some articles on the durability of chitosan-functionalized polyester [26], but few on cotton/polyester blended fabrics [25,29]. Due to the increasing requirements for durable chitosan functionalization over several maintenance cycles, many authors are researching how to bind chitosan to the textile substrate. In a previous work by the same authors [10], chitosan in the form of solid particles was well implemented during the mercerization process of cotton. In the second work [29], chitosan powder was dissolved in CA with SHP as a catalyst and applied to cotton and polyester/cotton fabric with an open cotton cellulose structure and hydrolyzed polyester by the jig–dry–cure process, which resulted in durable antimicrobial properties. Therefore, the aim of this research was to investigate the durability of chitosan functionalization of cellulosic textile substrates, cotton and cotton/polyester blended fabrics, using MA or BTCA as crosslinker and SHP as a catalyst.

## 2. Materials and Methods

### 2.1. Materials

Standard cotton fabric (CO) and polyester/cotton fabric (PES/CO) from WFK (wfk-Testgewebe GmbH, Brüggen, Germany) were used in this study. Standard cotton fabric (WFK label 10A) is defined in ISO 2267:1986 [41]. Polyester/cotton fabric (WFK label 20A) is manufactured to be as similar as possible to standard cotton fabric. The characteristics of the fabrics are as follows: CO—100% cotton; PES/CO—65% polyester/35% cotton; mass per unit area 170 g/m^2^; yarn count of warp and weft 27/27 cm^−1^ and linear density 295 dtex, canvas embroidery.

The chitosan provided by Tricomed SA (Lodz, Poland) was purchased from Mathani Chitosan Pvt. Ltd. (Veraval, Gujarat, India). The chitosan particle diameter range was 1 to 0.5 µm, the molecular weight (Mn) was 80, and the degree of deacetylation (DDA) 90. 

Maleic acid (MA) was purchased from Scharlau (Hamburg, Germany), 1,2,3,4-Butanetetracarboxylic acid (BTCA) and sodium hypophosphite monohydrate (SHP) from Sigma-Aldrich (Darmstadt, Germany), and NF-9 (nonionic surfactant with 9 ethylene oxide groups) from Kemo (Zagreb, Croatia).

Reference detergent 3 (ECE reference detergent 98), a non-phosphate powder detergent without optical brighteners for use according to ISO 6330:2021 [42] was purchased from SDC Enterprises Ltd. (Holmfirth, UK).

### 2.2. Chitosan Functionalization

Chitosan activation was performed using Maleic acid (MA) and 1,2,3,4-Butanetetracarboxylic acid (BTCA). Fabrics were aged for 20 h in a bath containing 15 g/L chitosan, 25 g/L MA or BTCA, 10 g/L SHP, and 2 g/L NF-9. After aging, fabrics were microwaved at 80 W for 5 min. The pad–dry–cure method was performed by squeezing on a padding machine (Benz, Zurich, Switzerland) with a wet pickup of 100%, followed by conductive drying at 100 °C for 2 min and thermocondensation at 150 °C for 3 min.

### 2.3. Maintenance Procedure

After chitosan functionalization, fabrics were subjected to 10 maintenance cycles. Washing was performed according to ISO 6330:2021 [42] with 20 g/L Reference detergent 3 at 60 °C in a Wascator FOM71 CLS machine (Electrolux, Stockholm, Sweden). Drying was performed in the T5130LAB drying machine (Electrolux, Stockholm, Sweden) according to Procedure F.

The labels and treatments are listed in Table 1.

### 2.4. Characterization Methods

The fabric properties were tested before and after functionalization (without additional rinsing) and after the 3rd and 10th maintenance cycles.

The characterization of the surface and chemical composition of cotton (CO) and cotton/polyester (CO_PES) fabrics was performed by Fourier transform infrared (FT-IR) spectroscopy using the attenuated total reflection (ATR) measurement technique (Perkin Elmer, software Spectrum 100S, Shelton, CT, USA). Four scans were carried out for each sample at a resolution of 4 cm^−1^ between 4000 and 380 cm^−1^.

Tensile properties of fabrics were determined using the TensoLab Strength Tester (Mesdan, Italy) according to ISO 13934-1:2013 [43]. The distance between clamps was 100 mm, the bursting speed was 100 mm/min, and pretension was 2 N. From the results of breaking force, mechanical damage was calculated according to ISO 4312:1989 [44]:

(1) $${U_{m}=\frac{F_{0}-F}{F_{0}}\cdot 100}_{\;\;\;\;\;\;[\%]}$$

where *U_m_* is mechanical damage (wear) [%], *F*_0_ is the breaking force of the start fabric [N], and *F* is the breaking force of the finished/washed fabric [N]. As the start fabric, standard CO or CO_PES fabric was used. Additionally, each sample was compared to its pair regarding the number of washing cycles.

Spectral remission was measured on a remission spectrophotometer Spectraflash SF 300 (Datacolor, Dietlikon, Switzerland). The whiteness degree according to CIE (W_CIE_) was calculated from remission in accordance with ISO 105-J02:1997 [45], and Yellowing Index (YI) in accordance to DIN 6167:1980 [46].

The antimicrobial activity was determined according to AATCC TM 147-2016 [47]. Activity was determined to be associated with Gram-positive bacteria *Staphylococcus aureus* ATCC 6538 (*S. aures*), Gram-negative bacteria *Escherichia coli* ATCC 8739 (*E. coli*), and microfungi—yeast *Candida albicans* ATCC 10231 (*C. albicans*).

## 3. Results and Discussion

The durability of chitosan functionalization of cellulosic fabrics, cotton (CO) and cotton/polyester blended fabrics (CO_PES), using maleic acid (MA) or 1,2,3,4-butanetetracarboxylic acid (BTCA) as a crosslinking agent and sodium hypophosphite monohydrate (SHP) as a catalyst, was studied. Physicochemical characterization was carried out after the functionalization of chitosan and after the 3rd and 10th maintenance cycles. 

**Figure 3:**
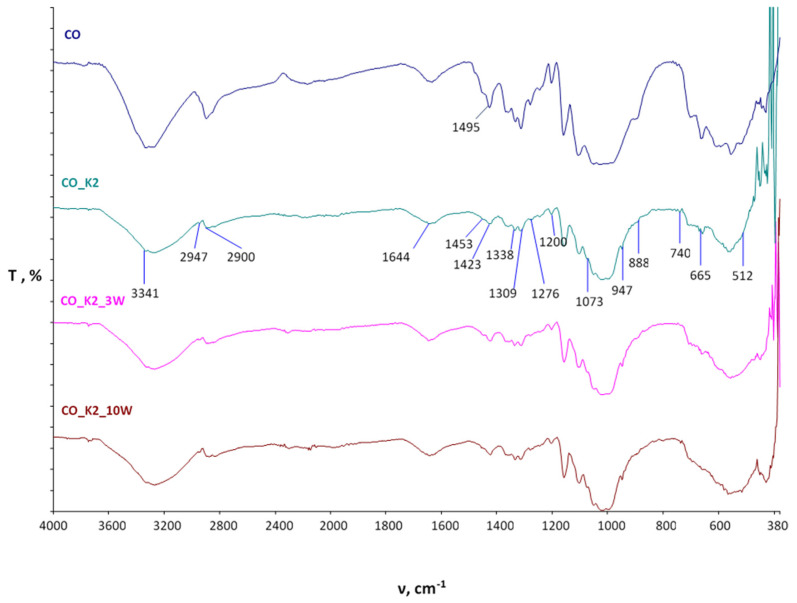
FTIR spectral bands of chitosan-functionalized cotton fabrics using MA as a crosslinking agent before and after maintenance cycles compared to untreated fabric.

**Figure 4:**
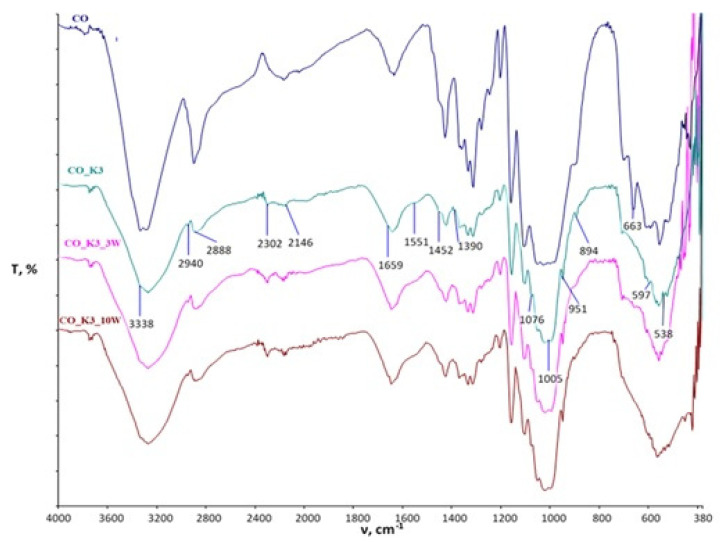
FTIR spectral bands of chitosan-functionalized cotton fabrics using BTCA as a crosslinking agent before and after maintenance cycles compared to untreated fabric.

**Figure 5:**
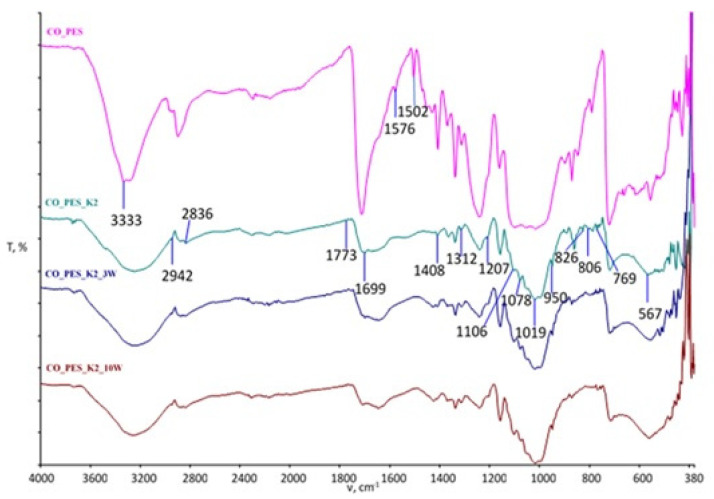
FTIR spectral bands of chitosan-functionalized cotton/polyester fabrics using MA as a crosslinking agent before and after maintenance cycles compared to untreated fabric.

**Figure 6:**
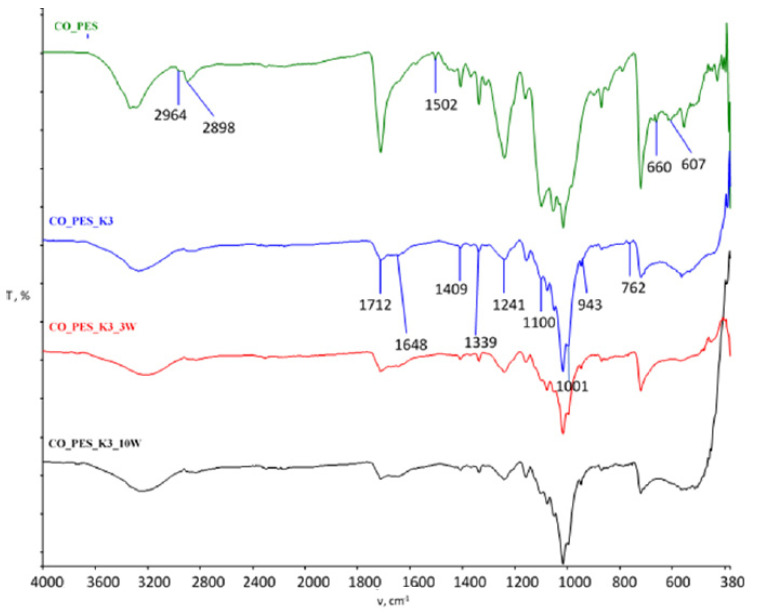
FTIR spectral bands of chitosan-functionalized cotton/polyester fabrics using BTCA as a crosslinking agent before and after maintenance cycles compared to untreated fabric.

The spectral curves obtained by FTIR-ATR analysis of the cotton fabric after functionalization and maintenance are shown in Figure 3 (MA) and Figure 4 (BTCA), and those of the cotton/polyester blended fabric are shown in Figure 5 (MA) and Figure 6 (BTCA).

Figure 3 shows the spectral bands of cotton fabrics functionalized with chitosan using MA as a crosslinking agent (K2) before and after maintenance cycles (3W, 10W) compared to untreated standard cotton fabric (CO). The spectral bands of the functionalized chitosan and the untreated cotton samples at 3341 cm^−1^ indicate the stretching of the -OH groups, which was changed in the chitosan-functionalized samples due to the overlap with the stretching of the N-H group in the chitosan in the same region, while at 1276 cm^−1^ bending of -OH occurred. The spectral bands of CO_K2 show the occurrence of symmetric and asymmetric vibrations within the C-H bonds characteristic of polysaccharides at wavenumbers 2947, 2900, and 888 cm^−1^. The peaks at 1644, 1338, and 1309 cm^−1^ are less intense in cotton fabric functionalized with chitosan than in untreated ones because of the stretching of the C=O bond within the cellulose. It can be assumed that the change in intensity is due to the presence of residual N-acetyl groups in the chitosan. At the wavenumber 1453 cm^−1^, a peak formed that was due to stretching within the CH_2_ group, while at 1423 cm^−1^, CH_3_ groups were present in addition to the CH_2_ group. The stretching of C-O groups is observed at wavenumbers 1073, 740, 665, and 512 cm^−1^. According to the literature, the peak at wavelength 947 cm^−1^ represents the binding of the P-O group to the trimethyl group, which may be present in the chitin structure. At the wavenumber 1495 cm^−1^, a sharp peak is seen in untreated standard cotton fabric, which is due to the bending of the -C-O-H group in the -OH plane and is much less pronounced after chitosan treatment [23,29,48,49,50,51].

Figure 4 shows the FTIR spectral bands of cotton fabrics functionalized with chitosan using BTCA as a crosslinking agent (K3) before and after maintenance cycles compared to untreated standard cotton fabric (CO). The spectral bands at 3338 cm^−1^ indicate stretching of the NH and OH groups as a result of hydrogen bonding between the groups, and at 1551 cm^−1^ indicate stretching of the N-H bond (secondary amide). The presence of C-H bonds and their stretching can be observed at 2940, 1005, and 894 cm^−1^. CH_3_ groups are present at wavenumbers 2888 and 1390 cm^−1^ and 1452 cm^−1^ together with CH_2_. The peak at 1659 cm^−1^ indicates C-O overlapping with NH_2_, reducing its intensity. Vibrations of the C-O bond are observed at 1076, 597, and 538 cm^−1^. The peak at 947 cm^−1^ indicates P-O vibrations in chitosan. At 663 cm^−1^, the bending of the -C-O-H group in the -OH plane can be observed in the CO sample [23,29,48,49,50,51].

All the physicochemical changes evident in the spectral bands of the treated samples after 3 and 10 maintenance cycles are clearly visible and indicate a constant change in the physicochemical properties of the treated samples.

The spectral bands of the cotton/polyester fabrics functionalized with chitosan using MA as the crosslinking agent before and after the maintenance cycles compared to the untreated fabric are shown in Figure 5 and for the cotton/polyester fabrics functionalized with chitosan using BTCA as the crosslinking agent in Figure 6. For the untreated CO_PES sample, a sharp peak is visible in the 1712 cm^−1^ wavenumber region corresponding to vibrations within the ester bonds. After treatment with chitosan, a small shift in wavenumber is seen in this peak, but also an extremely weak intensity of the peak in this region, indicating damping of the vibrations by the presence of chitosan. The resulting change is visible in all samples even after 3 and 10 maintenance cycles.

After chitosan functionalization, a new peak appears at 1699 cm^−1^ when MA was used and 1648 cm^−1^ when BTCA was used, indicating the stretching of amide I. Spectral bands at 2942 cm^−1^ indicate the occurrence of symmetric and asymmetric vibrations within the C-H bonds, while at 2836 cm^−1^ with this vibration, the stretching of the C-O group occurs, as well as at 1773, 1106 (1100), and 1019 cm^−1^. C-O groups are present at wavenumbers 1576, 826, 806, 769 (762), and 567 cm^−1^ in the untreated CO_PES fabric sample. C=O stretching confirming residual N-acetyl groups is visible at 1341 (1339) cm^−1^, as is the presence of C=O in the untreated fabric at 1312 cm^−1^. The peak at 1078 cm^−1^ indicates C-O bond stretching, as does the peak at 687 cm^−1^, indicating the presence of CO. The peak at 1408 (1409) cm^−1^ indicates the stretching of the N-H bond. Asymmetric CH_2_ stretching is visible at wavenumber 1339 cm^−1^, while C-H is visible at 868 cm^−1^ in the treated sample and 788 cm^−1^ in the untreated sample [23,29,48,49,50,51].

Based on the spectral bands obtained, it is clear that physicochemical changes occurred in all processed samples, as evidenced by the appearance of peaks, the decrease or increase in existing peaks, and the disappearance of individual peaks. All resulting changes are still visible after 10 maintenance cycles.

**Figure 7:**
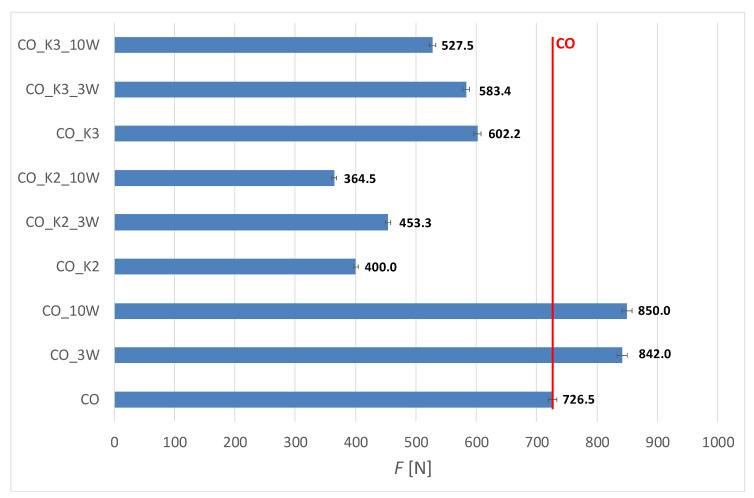
Breaking force, *F* [N], with an interval of confidence (IC99%) of cotton (CO) fabric before and after functionalization with chitosan and 10 maintenance cycles.

**Figure 8:**
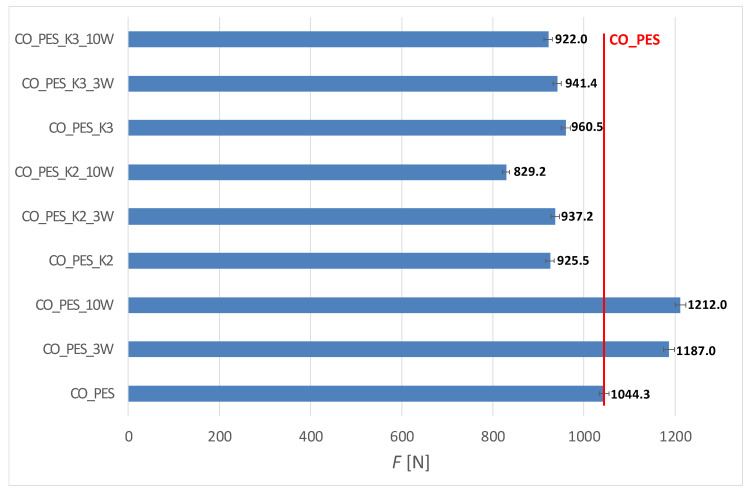
Breaking force, F [N], with an interval of confidence (IC99%) of cotton/polyester (CO_PES) fabric before and after functionalization with chitosan and 10 maintenance cycles.

The results of the mechanical properties of the fabric before and after functionalization with chitosan and 10 maintenance cycles, expressed as the average braking force (*F*) with a 99% confidence interval (IC99%), are shown in Figure 7 and Figure 8. The elongation at break (*ε*) and mechanical damage (*U_m_*), calculated according to ISO 4312:1989 [44] from the results of the breaking force with respect to the initial (untreated) fabric and with respect to the number of maintenance cycles, are shown in Table 2 and Table 3.

From the results of the mechanical properties, it appears that significant damage occurred in all processes of chitosan functionalization. The damage was mainly caused by the functionalization of cotton in an acidic medium [22]. Comparing the use of MA and BTCA as crosslinking and activating agents, it can be seen that the damage is much greater when maleic acid is used (for CO_K2, the strength loss is 45%, while for CO_K3 it is only 17%). With blended fabric, the damage is less because the polyester component retains strength (for CO_PES_K2, the strength loss is 11%, and for CO_K3, only 8%). The reason for this is the higher sensitivity of cotton to acid action compared to polyester, but also the greater number of free groups in the cotton through which the acid could have penetrated. In addition, all wet treatments cause swelling of the cellulosic materials, which causes the fabric to shrink after drying, resulting in increased yarn thickness and strength. However, for the cotton fabrics after chitosan functionalization using MA as a crosslinking agent (CO_K2_10W), the greatest mechanical damage was due to acid activity during functionalization and after mechanical agitation during the 10 maintenance cycles.

The whiteness degree according to the CIE (*W*) and Yellowing Index (*YI*) calculated from the spectral remission of cellulosic fabrics (CO and CO_PES) functionalized with chitosan after functionalization and maintenance cycles are shown in Table 4.

Table 4 shows the changes in whiteness and yellowing of the cellulosic fabrics functionalized with chitosan and after functionalization and maintenance. All fabrics yellowed after functionalization, and accordingly, their whiteness decreased. By performing three maintenance cycles, the whiteness of all the samples approached the whiteness of untreated fabric, and yellowing was decreased. After 10 maintenance cycles, the whiteness of the fabrics increased. Cotton fabric treated with 1,2,3,4-butanetetracarboxylic acid (CO_K3) resulted in great whiteness, so the yellowing index was almost 0.

**Figure 9:**
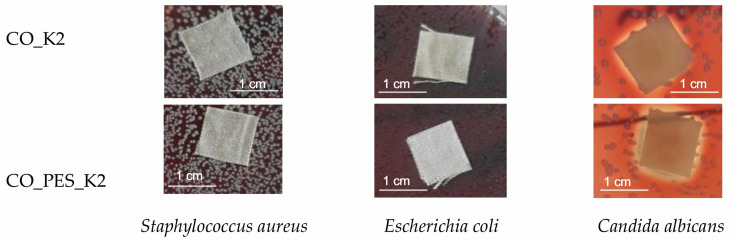
The antimicrobial activity of cellulosic fabrics after functionalization with chitosan and MA examples.

The results of antimicrobial activity determined according to AATCC TM 147-2016 are shown in Table 5. In Figure 9, exemplary photos of the antimicrobial activity of cellulosic fabrics after functionalization with chitosan and MA (K2) are shown.

From the results in Table 5, it can be seen that both untreated fabrics and CO and CO_PES showed no activity against Gram-positive bacteria *Staphylococcus aureus*, Gram-negative bacteria *Escherichia coli*, and microfungi *Candida albicans*. However, it can be seen that treatment with chitosan led to some antimicrobial activity. Both fabrics showed good activity against the microfungus *Candida albicans*. Although the zone of inhibition can be observed, there are no bacterial colonies directly under the sample in the contact area, so the fabrics show antimicrobial activity. Activity against bacteria was not observed. The antimicrobial activity of chitosan is attributed to its polycationic nature. It most likely interacts with the anionic membrane of the microorganism, resulting in a change in permeability that causes cell death by leakage of intracellular plasma. Unlike CA [29], MA and BTCA as cross-linking agents lead to better binding of chitosan to cellulose, so the electropositive character of chitosan plays a lesser role and has fewer active sites for interaction with bacteria.

It should be emphasised that the antimicrobial activity obtained against the microfungus *Candida albicans* is maintained after 10 maintenance cycles, indicating a sufficient amount of chitosan in the fabric structure.

## 4. Conclusions

In this work, the durability of chitosan finishing of cellulosic textile substrates, cotton and cotton–polyester blended fabrics, was studied. Maleic acid (MA) and 1,2,3,4-butanetetracarboxylic acid (BTCA) were used as crosslinking and chitosan-activating agents, and sodium hypophosphite monohydrate was used as a catalyst for chitosan functionalization. To determine the durability of the functionalization, the fabrics were subjected to 3 and 10 maintenance cycles. Both acids showed good crosslinking properties, regardless of the cellulosic fabric (cotton or cotton–polyester blend). The mechanical damage of the cotton fabric was greater than that of the cotton–polyester blend, which was due to the sensitivity of cotton to acids compared to polyester, but also to the greater number of free groups over which chitosan crosslinking could occur. Maleic acid caused a higher percentage of mechanical damage compared to 1,2,3,4-butanetetracarboxylic acid as a crosslinking agent, regardless of concentration. Maleic acid and 1,2,3,4-butanetetracarboxylic acid were found to be good, environmentally friendly crosslinking agents, as cotton or cotton–polyester blended fabrics showed good durability even after 10 maintenance cycles. Activity against bacteria was not observed, but activity against the microfungus *Candida albicans* was present even after 10 maintenance cycles. Compared to maleic acid, BTCA proved to be a better crosslinking agent for chitosan, considering all tested properties.

## Figures and Tables

**Table 1:** The labels and treatments of cellulosic fabrics.

Label	Fabric Description
CO	Cotton standard fabric (WFK label 10A)
CO_PES	Cotton/polyester blended fabric (WFK label 20A)
_K2	Chitosan functionalized cellulosic fabric with MA as crosslinking agent
_K3	Chitosan functionalized cellulosic fabric with BTCA as crosslinking agent
_3W	Chitosan functionalized cellulosic fabric after 3 maintenance cycles
_10W	Chitosan functionalized cellulosic fabric after 10 maintenance cycles

**Table 2:** Elongation at break (*ε* [%]) and mechanical damage (*U_m_* [%]) of cotton fabrics calculated related to the start fabric and to its pair related to the number of maintenance cycles.

Label	*ε* [%]	*U_m_* [%]	*U_m related pair_* [%]
CO	14.90	0.00	-
CO_3W	17.70	−15.90	-
CO_10W	16.65	−17.00	-
CO_K2	18.80	44.94	44.94
CO_K2_3W	20.88	37.60	46.16
CO_K2_10W	18.10	49.83	57.12
CO_K3	18.15	17.11	17.11
CO_K3_3W	18.60	19.70	30.72
CO_K3_10W	17.70	27.39	37.94

**Table 3:** Elongation at break (*ε* [%]) and mechanical damage (*U_m_* [%]) of cotton/polyester blended fabrics calculated related to the start fabric and to its pair related to the number of maintenance cycles.

Label	*ε* [%]	*U_m_* [%]	*U_m related pair_* [%]
CO_PES	14.90	0.00	-
CO_PES_3W	17.70	−13.66	-
CO_PES_10W	16.65	−16.06	-
CO_PES_K2	18.80	11.38	11.38
CO_PES_K2_3W	20.88	10.26	21.05
CO_PES_K2_10W	18.10	20.60	31.58
CO_PES_K3	18.15	8.03	8.03
CO_PES_K3_3W	18.60	9.86	20.69
CO_PES_K3_10W	17.70	11.71	23.93

**Table 4:** The whiteness degree according to the CIE (*W*) and Yellowing Index (*YI*) of cellulosic fabrics (CO and CO_PES) functionalized with chitosan after functionalization and maintenance cycles.

Fabric	CO_		CO_PES_	
	*W*	*YI*	*W*	*YI*
_	73.4	4.37	74.8	3.28
_K2	49.0	12.38	60.9	8.16
_K2_3W	73.8	4.29	70.1	5.14
_K2_10W	77.8	2.62	78.9	2.01
_K3	48.2	12.94	54.6	10.66
_K3_3W	73.5	4.47	67.4	6.15
_K3_10W	83.5	0.83	70.6	4.99

**Table 5:** The results of antimicrobial activity of cellulosic fabrics before and after functionalization with chitosan and 10 maintenance cycles.

Fabric	*Staphylococcus aureus*		*Escherichia coli*		*Candida albicans*	
	CO_	CO_PES_	CO_	CO_PES_	CO_	CO_PES_
_	−	−	−	−	−	−
_K2	−	−	−	−	+/−	+/−
_K2_3W	−	−	−	−	+/−	+/−
_K2_10W	−	−	−	−	+/−	+/−
_K3	−	−	−	−	+/−	+/−
_K3_3W	−	−	−	−	+/−	+/−
_K3_10W	−	−	−	−	+/−	+/−

+ antimicrobial activity (zone of inhibition can be observed); +/− antimicrobial activity (no colonies beneath); − no antimicrobial activity.

## Data Availability

Data are available in a publicly accessible repository.
